# The impact of the perception of treatment choice on satisfaction with treatment, experienced chemotherapy burden and current quality of life

**DOI:** 10.1038/sj.bjc.6601903

**Published:** 2004-05-25

**Authors:** S J T Jansen, W Otten, C J H van de Velde, J W R Nortier, A M Stiggelbout

**Affiliations:** 1 Department of Medical Decision Making, J10S, Leiden University Medical Center, PO Box 9600, 2300 RC Leiden, The Netherlands

**Keywords:** quality of life, treatment choice, cancer, adjuvant chemotherapy

## Abstract

Previous research has shown that involving patients in the decision-making process may improve their quality of life (QoL). Our purposes were to assess: (1) whether early-stage breast cancer patients perceived that they had treatment choice with regard to adjuvant chemotherapy, (2) what reasons patients provide for their perception of having had no choice of treatment and (3) whether the perception of treatment choice is related to satisfaction with the assigned treatment, experienced chemotherapy burden and current QoL. A total of 448 patients, treated between 1998 and 2003, filled in the questionnaire (response rate: 62%). Patients who indicated that they had not perceived a choice regarding chemotherapy could tick off one or more reasons out of 10 reasons, or provide their own reason(s). Quality of life was measured on a Visual Analogue Scale, by means of the EuroQol, and by means of the Hospital Anxiety and Depression Scale. Of the 405 patients who had answered the question on treatment choice, 316 patients (78%) had perceived no choice. The most frequently indicated reason for lack of choice was: ‘I follow the doctor's advice.’ We found no differences in the levels of satisfaction with assigned treatment and in how much of a burden they found chemotherapy between patients who perceived a choice of treatment and those who did not. In general, the perception of choice seemed to have no impact on QoL. However, we found an interaction effect, which indicated that the impact of perception of treatment choice on QoL was dependent upon whether the patient had been treated with chemotherapy or not. Within the group of patients who had not been treated with chemotherapy, the perception of having had a choice was related to lower current QoL. In cases when the decision to be treated or not has potential consequences for the chance of survival, patients' QoL may not be improved by the perception of having had a choice of treatment.

Over the years, health care providers have moved from a more paternalistic approach to one that actively encourages patient autonomy and shared decision-making. The involvement of patients in making decisions about their own care may contribute to medical care that is in harmony with patients' wants and needs, and may perhaps improve health outcomes. Patients who perceive that they have decisional control in treatment decision-making may regain a sense of control and mastery over their disease or treatment (Morris and Royle, 1988; Hack *et al*, 1994; Street and Voigt, 1997; Deadman *et al*, 2001), which may ultimately lead to a higher quality of life (QoL) (Morris and Royle, 1988; Street and Voigt, 1997; Deadman *et al*, 2001; Polsky *et al*, 2002; Mandelblatt *et al*, 2003). For example, Polsky *et al* (2002) observed that patients who believed they had had a choice of surgical treatment for breast cancer reported higher QoL scores by means of a Visual Analogue Scale at 5 months following surgery, than did patients who perceived themselves to have had no control over the decision. Similarly, Mandelblatt *et al* (2003) concluded that breast cancer survivors, who felt that they had had no choice of treatment, reported significantly less general satisfaction, more bodily pain and lower mental health scores, 3–5 years after their primary treatment.

Much research has been carried out into the impact of offering choice of treatment in the domain of surgery for breast cancer, because the majority of breast cancer patients have the option of choosing between modified radical mastectomy and breast-conserving therapy. Both treatments are equally effective, but may be valued differently by patients. A number of studies showed that breast cancer patients who had actually been offered a choice of surgery had a better sense of psychological well being than patients who had not been given a choice based on clinical arguments, such as central position of the tumour, inadequate tumour-to-breast ratio and multiple tumours (Morris and Royle, 1988; Al-Ghazal *et al*, 2000; Deadman *et al*, 2001). Other studies observed similar differences in both physical and psychological well being, between patients who felt that they had had a choice of treatment and patients who believed they had had no choice of treatment, irrespective of whether the choice had actually been offered (Street and Voigt, 1997; King *et al*, 2000; Polsky *et al*, 2002; Mandelblatt *et al*, 2003). It seems that the subjective experience of control over decisions is as important for patients' QoL as actually being offered a choice.

As well as improving the QoL, offering cancer patients the opportunity to participate in making decisions regarding their treatment may also increase satisfaction with treatment and care received. Gattellari *et al* (2001) observed that patients who perceived that they had shared the treatment decision with their physician were significantly more satisfied with the consultation, the amount of information and the emotional support received from their physician, than patients who perceived the treatment decision as having been made exclusively by themselves or their physician. Benefits that have been observed in patients with diseases other than cancer include: less stress, decreased levels of symptom distress and concern about illness, increased functional status, coping, control over illness, self-efficacy, understanding of and commitment to the treatment plan and satisfaction with their physician (Davison *et al*, 1995). Krupat *et al* (2000) observed that, in the case of surgical patients, more perception of control was related to more satisfaction with the care that patients had received during their hospital stay.

In general, research has been carried out into patients' perceptions of having had a choice of treatment in the case of two equally effective treatments, such as mastectomy and breast-conserving therapy. However, we believe that involving patients in making decisions about treatment may also be important when deciding between treatments that are not equally effective and which may differ with regard to the values that patients attach to the various consequences of each option. For example, decisions about treatment with adjuvant chemotherapy imply a complex and difficult trade-off between increased probability of survival and deterioration in QoL, due to the side effects of the treatment. All patients will experience the side effects, but only some patients will actually benefit from this treatment. Patients' preferences regarding this trade-off have shown to vary widely (e.g., Jansen *et al*, 2001) and to differ from the preferences of doctors (e.g., Slevin *et al*, 1990). Charles *et al* (1997) stress the importance of shared decision-making in the case of adjuvant chemotherapy, because: (1) several treatment options exist with different possible outcomes and substantial uncertainty, (2) there is often no clear-cut right or wrong answer and (3) the impact of the treatment on the patient's physical and psychological well being will vary. The choice between two equally effective treatments, such as breast-conserving therapy and mastectomy, may have other implications for well being than the choice between a treatment that has the potential to increase the chance of being cured at the costs of side effects, or no treatment. As far as we know, in the case of adjuvant chemotherapy, there are no studies that have examined the perception of freedom in choice of treatment and its consequences for QoL and satisfaction with treatment.

The purposes of this study were to assess: (1) whether early-stage breast cancer patients perceived a choice of treatment with regard to adjuvant chemotherapy, (2) what reasons patients provide for having perceived no choice of treatment and (3) whether perception of treatment choice is related to satisfaction with the assigned treatment, the amount of chemotherapy burden experienced and current QoL.

## PATIENTS AND METHODS

### Patients

Patients with early-stage breast cancer, who had (part of) their primary treatment in the Leiden University Medical Center between January 1998 and January 2003, received a letter of invitation in which the study was explained. The exclusion criteria were: metastasised disease and poor understanding of the Dutch language. Patients could indicate on a reply sheet whether they wanted to receive the questionnaire and could return this sheet in a prepaid envelope. The questionnaire and a prepaid envelope were sent to patients who responded positively to our request. Patients who did not reply received one reminder.

### Methods

Perceived treatment choice was measured by asking: ‘Do you feel that you had a choice regarding treatment with adjuvant chemotherapy? (yes/no).’ This question followed a question asking whether the patient had been treated with adjuvant chemotherapy. Patients whose perception was that they had had no treatment choice were asked to tick off one or more reasons out of 10, or provide their own reason(s) for this perception. The exact formulation of the statements is given in Table 2. Note that only four of the 10 reasons were exactly the same for patients who had undergone chemotherapy and those who had not. This is because some reasons do not apply to treatment with chemotherapy, for example, ‘In my case chemotherapy is no use’, whereas others refer only to treatment with chemotherapy. The 10 reasons were formulated on the basis of prior qualitative research into the determinants of patients' preferences for adjuvant chemotherapy and perceived freedom of treatment choice (Jansen *et al*, 2003).

Satisfaction with the assigned treatment was investigated by asking, ‘Are you satisfied with the fact that you have (not) been treated with chemotherapy?’. Patients could indicate their response on a five-point rating scale, anchored by ‘very dissatisfied’ (1) and ‘very satisfied’ (5). The experienced chemotherapy burden was explored by asking, ‘What is your experience of chemotherapy treatment?’ and providing a five-point response scale, anchored with ‘very difficult’ (1) and ‘very easy’ (5).

Current QoL was measured on (1) a Visual Analogue Scale, ranging from 0 ‘death’ to 1 ‘perfect health,’ (2) by means of the EuroQol utility scores (Dolan, 1997) and (3) by means of the Hospital Anxiety and Depression Scale (HADS, Dutch version: Spinhoven *et al*, 1997). The EuroQol utility score is derived by weighing patients' responses to five questions in various domains of QoL (mobility, self-care, usual activities, pain/discomfort and anxiety/depression). The resulting utility scores range from −1 ‘worst QoL’ to 1 ‘best QoL’. The HADS was originally developed by Zigmund and Snaith (1983) and has two subscales: anxiety and depression. Each subscale contains seven questions that have four response categories (0–3). The score for each subscale can range from 0 to 21, with higher scores indicating more anxiety or depression.

### Analysis

Patient and clinical characteristics are shown by means of descriptive methods. Differences in patient and clinical characteristics between responders and nonresponders, and between patients who perceived that they had a choice of treatment and those who did not, were analysed by means of *χ*^2^ statistics (gender, marital status, education, type of cancer and having experienced chemotherapy) and independent samples *t*-test (age, time passed since surgery until filling in questionnaire, time passed since completion of chemotherapy until filling in questionnaire).

The impact of perceived treatment choice on satisfaction with assigned treatment and on experienced chemotherapy burden was analysed by means of univariate analysis of variance. The impact of perceived treatment choice on the current QoL was analysed by means of multivariate analysis of variance. Visual Analogue Scale scores, EuroQol scores, HADS Depression and HADS Anxiety scores were included simultaneously as dependent variables in this analysis. Perception of treatment choice was included as a between-group factor in all models. Patient and clinical characteristics that turned out to be related to the perception of treatment choice were to be included as potential confounders in the analyses, either as a between-group factor (categorical variables), or as a covariate (continuous variables).

## RESULTS

### Description of the patient group

Between February and April 2003, 719 patients were asked to participate in the study. In all, 102 patients (14%) indicated that they did not want to receive the questionnaire, five patients (1%) were deceased, four patients were not eligible due to dementia (as indicated by their family), six patients had moved, and 72 patients (10%) did not respond at all. A total of 530 patients (74%) indicated that they wanted to receive the questionnaire. In all, 448 questionnaires were returned (85% of 530; 62% of 719).

Characteristics of the 448 patients who returned the questionnaire and the 271 patients who did not fill in the questionnaire are presented in Table 1

**Table 1:** Patient and clinical characteristics of responders and nonresponders

	**Responders (*n*=448)**	**Nonresponders (*n*=271)**
*Age*		
Mean (s.d.)	60 (11)	63 (12)
Range	32–89	34–90
		
*Gender*		
Female	445 (99%)	268 (99%)
		
*Having experienced adjuvant chemotherapy*		
Yes	171 (38%)	71 (26%)

. There were no differences between the responders and nonresponders with regard to gender. However, the nonresponders were slightly older and had experienced adjuvant chemotherapy less frequently (both: *P*<0.01).

### Perception of treatment choice

Of the 405 (90% of 448) patients who had answered the question about perception of treatment choice, 316 patients (78%) responded that they had perceived a lack of choice regarding treatment with adjuvant chemotherapy. Characteristics of the two patient groups (choice *vs* no choice) are presented in Table 2

**Table 2:** Patient and clinical characteristics of patients who perceived treatment choice and those who did not (*n*=405)

	**Choice (*n*=89)**	**No-choice (*n*=316)**
*Age*		
Mean (s.d.)	57 (11)	59 (10)
Range	36–89	32–85
		
*Gender*		
Female	88 (99%)	314 (99%)
		
*Marital status*		
Married/living (apart) together	65 (73%)	243 (77%)
Widowed/single/divorced	24 (27%)	73 (23%)
		
*Education*		
<10 years	44 (50%)	182 (59%)
10–15 years	23 (26%)	66 (21%)
>15 years	21 (24%)	62 (20%)
		
*Time from surgery to filling in questionnaire*		
Mean number of days (s.d.)	1118 (425)	1041 (437)
Range	337–1935	161–1923
		
*Having experienced adjuvant chemotherapy*		
Yes	56 (63%)	117 (37%)
		
*Time from completion of chemotherapy to filling in questionnaire*	(*n*=56)	(*n*=117)
Mean number of days (s.d.)	1019 (422)	918 (354)
Range	332–2397	93–1853

. There were no differences in perception of treatment choice with regard to gender (*P*=0.63), marital status (*P*=0.45), education (*P*=0.35), time passed since surgery until filling in the questionnaire (*P*=0.14) and time passed since completion of chemotherapy until filling in the questionnaire (*P*=0.12). However, patients who had perceived a treatment choice had more often been treated with adjuvant chemotherapy (*P*<0.01). Furthermore, we observed a trend that patients who had perceived treatment choice were slightly younger (*P*=0.08). Thus, having had adjuvant chemotherapy and age will be included in the analyses of variance, because they are potential confounders of the relationship between the perception of treatment choice and the dependent variables.

An overview of the reasons for having the perception that there was no choice of treatment is provided in Table 3

**Table 3:** Reasons for the perception of lack of treatment choice

**Reason**	***n***	**%**	**Reason**	***n***	**%**
*Chemotherapy group* (*n*=*116*)			*No*-*chemotherapy group* (*n*=*198*)		
I follow the doctor's advice.	100	86%	I follow the doctor's advice.	112	57%
I'll do anything to be cured.	87	75%	Chemotherapy is not necessary.	93	47%
If it's got to be done, than it's got to be done.	60	52%	I do not need chemotherapy in order to be cured.	89	45%
It's to make sure.	54	47%	If it's not necessary, I would prefer not to have chemotherapy.	79	40%
Chemotherapy is necessary.	42	36%	I let my doctor decide.	32	16%
Doing nothing is no choice.	33	28%	Nobody is going to ask for chemotherapy.	20	10%
There is no alternative to chemotherapy.	33	28%	In my case chemotherapy is no use.	12	6%
I let my doctor decide.	10	9%	I haven't had time to think about it.	8	4%
I haven't had time to think about it.	10	9%	I don't ever want to have chemotherapy.	5	3%
Fate decided.	7	6%	Fate decided.	3	2%
Other reasons	15	13%	Other reasons	9	5%

. In the no-chemotherapy group, the mean number of checked reasons was 2.33 (s.d.=1.40; range 1–8) and in the chemotherapy group 3.89 (s.d.=1.84; range 1–10). Both in the group of patients who had been treated with adjuvant chemotherapy and in the group who had not, the most frequently indicated reason for the perception of lack of choice was: ‘I follow the doctor's advice’ (chemotherapy group: 86%, no-chemotherapy group: 57%). The second most important reason was ‘I'll do anything to be cured’ in the chemotherapy group (75%) and ‘Chemotherapy is not necessary’ in the no-chemotherapy group (47%).

### Relationship between perception of treatment choice, satisfaction with assigned treatment and experienced chemotherapy burden

In general (*n*=407), the mean score for satisfaction with the assigned treatment is 4.53 (±0.76), which indicates that patients were quite satisfied with their assigned treatment. The univariate analysis showed a significant effect (*P*<0.01) for (not) having experienced chemotherapy and for age (*P*=0.01), but not for perception of choice regarding treatment (*P*=0.78), or for the interaction between perception of treatment choice and having experienced adjuvant chemotherapy (*P*=0.56). The main effect for age indicates that older patients were more satisfied with their assigned treatment. Furthermore, patients who have undergone chemotherapy (*n*=170) are less satisfied with their assigned treatment than patients who have not been treated with chemotherapy (*n*=226) (mean satisfaction scores after correction for age: 4.33 *vs* 4.67). The perception of lack of treatment choice has no impact on satisfaction with assigned treatment.

The mean score for experienced chemotherapy burden is 2.66 (±1.06, *n*=175), which means that, in general, patients had found chemotherapy moderately difficult. The univariate analysis showed no effect of either perceived treatment choice (*P*=0.30) or age (*P*=0.11). Thus, experienced chemotherapy burden is not influenced by the perception of no treatment choice.

### Relationship between perception of treatment choice and current QoL

The mean current QoL scores are presented in Table 4

**Table 4:** Mean current quality of life scores for Visual Analogue Scale, EuroQol, HADS Anxiety and HADS Depression (*n*=361)

**Current quality of life**	**Choice**	**No choice**	**Effects**
Overall results:			Perceived choice: *P*=0.40
			Chemotherapy: *P*=0.54
			Interaction: *P*=0.12
			Age: *P*<0.01
			
*Univariate results:*			
Visual Analogue Scale^a^			
Chemo	0.77	0.75	Perceived choice: *P*=0.28
	(*n*=54)	(*n*=105)	Chemotherapy: *P*=0.76
No chemo	0.69	0.77	Interaction: *P*=0.01
	(*n*=28)	(*n*=174)	Age: *P*=0.02
			
EuroQol^a^			
Chemo	0.84	0.82	Perceived choice: *P*=0.19
	(*n*=54)	(*n*=105)	Chemotherapy: *P*=0.57
No chemo	0.74	0.83	Interaction: *P*=0.04
	(*n*=28)	(*n*=174)	Age: *P*=0.01
			
HADS anxiety^b^			
Chemo	4.37	4.87	Perceived choice: *P*=0.35
	(*n*=54)	(*n*=105)	Chemotherapy: *P*=0.10
No chemo	5.93	4.53	Interaction: *P*=0.05
	(*n*=28)	(*n*=174)	Age: *P*=0.18
			
HADS depression^b^			
Chemo	2.46	2.42	Perceived choice: *P*=0.06
	(*n*=54)	(*n*=105)	Chemotherapy: *P*=0.18
No chemo	4.39	2.87	Interaction: *P*=0.08
	(*n*=28)	(*n*=174)	Age: *P*<0.01

a A higher score refers to better quality of life.

b A lower score refers to less anxiety and depression.

. The multivariate analysis (overall results) showed a main effect for age (*P*<0.01), but no main effects for perceived treatment choice or having experienced adjuvant chemotherapy. There was a trend towards an interaction effect between perceived treatment choice and having experienced chemotherapy (*P*=0.12). The main effect for age indicates that, in general, older patients have a lower QoL. The interaction effect means that the impact of perception of treatment choice on current QoL is dependent upon experience with chemotherapy.

The univariate tests showed a main effect for age for all QoL instruments, except for HADS Anxiety. Furthermore, an interaction effect between perception of treatment choice and (not) having experienced chemotherapy was observed for all QoL instruments (VAS, EuroQol, HADS Anxiety: *P*⩽0.05, HADS depression: *P*=0.08).

The interaction effect of perceived choice and having experienced chemotherapy pointed in the same direction for all QoL instruments, indicating that, within the group of patients who had perceived a treatment choice, not being treated with chemotherapy was related to lower current QoL. In contrast, among the patients who had not perceived a treatment choice, not being treated with chemotherapy was related to higher current QoL scores.

To investigate this interaction effect in more detail, we repeated the analysis using simple main effects (perceived choice within chemo/no chemo group) and again including age as a covariate. In the group of patients who had experienced chemotherapy, we found no overall effect of perceived choice on QoL (*P*=0.58). Furthermore, none of the univariate effects for each of the QoL instruments reached statistical significance. In the patient group who had not been treated with chemotherapy, the overall effect of perceived choice reached borderline significance (*P*=0.07). The univariate results showed significant effects of perceived choice for the VAS (*P*<0.01), the EuroQol (*P*=0.02) and for HADS depression (*P*=0.05). No effect was observed for HADS anxiety (*P*=0.21). Interestingly, patients who had perceived a treatment choice experienced worse current QoL.

## DISCUSSION

### Perception of (no) treatment choice

Our first research goal was to explore whether breast cancer patients perceived freedom of choice regarding treatment with adjuvant chemotherapy. We found that 68% of patients, who had undergone chemotherapy, and 86% of patients, who had not experienced chemotherapy, reported that they had felt a lack of choice in treatment decision-making. We believe that these percentages are remarkably high, but we are not aware of previous studies regarding the choice for adjuvant chemotherapy with which we could compare our results.

In both treatment groups, the most frequently indicated reason for having experienced a lack of treatment choice was, ‘I follow the doctor's advice.’ Previous research has shown that the influence of specialist preferences on treatment choice may be considerable (Stoevelaar *et al*, 1999), even to the extent that the strongest predictor for the treatment decisions of patients with a life-threatening illness is the physician's treatment recommendation (Siminoff and Fetting, 1991; Smitt and Heltzel, 1997; Nold *et al*, 2000). In the study by Nold *et al* (2000), women with breast cancer reported that the surgeon was the most influential person in their treatment decision for breast-conserving therapy and more important than the fear of breast cancer or its recurrence. Gurmankin *et al* (2002), in a study using hypothetical treatment choices, observed that respondents are willing to follow the doctor's recommendation even if this goes against what is best with regard to maximising health and against what they would otherwise prefer. The results of our study suggest that the effect of the specialist's recommendation may be so strong that patients perceive that they have no choice but to follow the specialist's advice.

The results also show that 75% of patients who had undergone chemotherapy reported that their wish to do anything to be cured was a reason for the perception of having had no choice of treatment. In comparison, almost half of the patients in the no-chemotherapy group thought that chemotherapy was unnecessary and not needed to be cured, presumably because they believed that their doctors would otherwise have recommended this treatment. Thus, when stating that they have not perceived a treatment choice, patients may mean other things besides being offered choice of treatment by their doctor. These results are in agreement with the observations by Charles *et al* (1998), who describe in a qualitative study that many women were preoccupied with avoiding the possibility of disease recurrence. For this reason, these women believed that the only decision they could make was to accept the treatment that was offered. Some women felt that they had no choice but to undergo all relevant treatments available in order to reassure themselves that they had done everything possible to ‘fight’ their cancer. Furthermore, Charles *et al* reported that breast cancer patients believed that the options of adjuvant chemotherapy, or no adjuvant chemotherapy, were not of equal value. As these options were frequently perceived as ‘doing something’ *vs* ‘doing nothing’, patients felt that their illness gave them no choice but to undergo treatment. In our study, 28% of the patients who had undergone chemotherapy indicated that this reason played a role in their perception of no choice of treatment.

### Impact of perception of treatment choice on satisfaction with assigned treatment, experienced chemotherapy burden and current QoL

Contrary to expectation, perception of lack of treatment choice had no consequences for the level of satisfaction with assigned treatment. Similarly, the perception of no treatment choice had no consequences for experienced chemotherapy burden.

We found that, within the group of patients who had been treated with adjuvant chemotherapy, perception of treatment choice was not related to QoL. Furthermore, among the patients who had not undergone adjuvant chemotherapy, the perception of no treatment choice was related to higher QoL. These results are in contradiction with the results observed in other studies. For example, Mandelblatt *et al* (2003) observed that breast cancer patients' perceptions of no treatment choice were related to less general satisfaction, more bodily pain and lower mental health scores, 3–5 years post-treatment. Street and Voigt (1997) observed that in early-stage breast cancer patients, perceived choice was related to better QoL in the domain of physical well being (*r*=0.27, *P*<0.05) and functional well being (*r*=0.28, *P*<0.05) during the 12 months following breast surgery. Furthermore, Polsky *et al* (2002) concluded that perceived choice was related to better QoL measured by means of the VAS (*P*=0.03). The latter studies did not observe differences in the perception of treatment choice and its impact on QoL, between patients who had been treated by means of a mastectomy and patients who had undergone breast-conserving therapy. In the study by Mandelblatt *et al* (2003), the potential impact on QoL of an interaction effect between treatment undergone and perceived choice was not assessed.

However, the results of these studies were not straightforward. Mandelblatt *et al* (2003) found no effect of the perception of lack of treatment choice in the domains of: physical functioning, physical role, general health, emotional role, vitality and satisfaction with medical care. Street and Voigt (1997) did not observe a relationship between the perception of no treatment choice and emotional and social well being. Polsky *et al* (2002) did not find an impact of the perception of no treatment choice on QoL beyond 5 months after surgery when using the Visual Analogue Scale, and at none of the measurement points (5 months, 1 year and 2 years following surgery) when employing the Health Utilities Index (*P*=0.10). These results indicate that the effect of the perception of no treatment choice on QoL may differ, depending on, for example, the domain of QoL that is studied, the measurement instrument that is used, the treatment decision under concern, and the time passed between treatment decision and QoL assessment. In view of these results and the results observed in our study, we may conclude that the impact of the perception of no treatment choice on QoL seems rather modest and deserves further research.

If we adhere to the concepts of patient autonomy and shared decision making, we may consider the perception of no treatment choice as a negative outcome of treatment decision-making. However, it may be naive to assume that all patients want, and benefit from, active involvement in treatment decision-making (Fallowfield, 1997). The nature of the risks and benefits involved in different treatment options may influence the preferred involvement in treatment decision-making. The choice for adjuvant chemotherapy may have severe consequences because it may influence overall survival. Furthermore, the side effects of treatment could be considerable. In such situations, patients may be more inclined to hand over the treatment decision to their doctor. In such cases, patients' QoL may not be improved by the perception of having had a choice of treatment. In our study, patients who reported that they had experienced a choice of treatment and who had not been treated with chemotherapy consistently showed the lowest current QoL scores. A potential explanation for their low QoL scores may be that they feel very responsible for their choice not to be treated with chemotherapy and that they worry about the possibility of the disease recurring and of having made the wrong treatment decision.

### Limitations

As the nonresponders in our study were slightly older and had been treated with chemotherapy less frequently, we may not be able to generalise our results so that they refer to the whole population of disease-free patients treated for early-stage breast cancer within the past 5 years.

Another limitation of our study is that we do not know whether patients had actually been offered a choice of treatment. Furthermore, patients may have been provided with a choice, but may not have experienced it as such because of difficulties with the comprehension of information due to psychological distress (e.g. anxiety, depression) or specific coping strategies (e.g. denial). For example, Keating *et al* (2003) observed that, in one-third of cases, patients and surgeons disagreed about whether both breast-conserving therapy and mastectomy had been discussed. Patients reported more often than surgeons that only one of both treatments had been discussed.

However, we believe that the subjective experience of control over decisions is as important, and perhaps even more important for patients' QoL as having actually being offered a choice. Our interest lies in the impact of the subjective experience of having had (no) treatment choice, because this is what the patient is left with once the treatment decision has been made. Independent of whether or not treatment choice has actually been offered, the subjective experience is what the patient remembers, and it seems likely that it is this subjective experience that determines the impact on QoL and satisfaction rather than having had objective choice. In support of this hypothesis, a number of studies have indeed shown that patients who had perceived a choice of treatment had better psychological and physical well being than patients who had not perceived a choice, irrespective of whether the choice had actually been offered (Street and Voigt, 1997; King *et al*, 2000; Polsky *et al*, 2002; Mandelblatt *et al*, 2003).

## CONCLUSION

Using a large sample of patients, treated for early-stage breast cancer within the past 5 years, we have shown that, in general, patients' perceptions of no choice of adjuvant chemotherapy did not have an impact on their satisfaction with assigned treatment, their experienced chemotherapy burden and their current QoL. However, patients' perception of having had a treatment choice may have negative consequences for current QoL scores in the case when patients have not undergone chemotherapy. This may be due to the fact that the decision about chemotherapy may have severe consequences for both survival and QoL.
